# Global accreditation practices for accelerated medically trained clinicians: a view of five countries

**DOI:** 10.1186/s12960-021-00646-4

**Published:** 2021-09-14

**Authors:** James Antwi, Anthony Asare Arkoh, Joseph Kiprop Choge, Turi Woticha Dibo, Alias Mahmud, Enkhtuya Vankhuu, Erick Kizito Wanyama, Danette Waller McKinley

**Affiliations:** 1 Centre for Health and Social Policy Research, West End University College, Accra, Ghana; 2 Department of Medicine, Graduate Physician Assistants Association of Ghana, Rock Hospital, Accra, Ghana; 3 Clinical Medicine Department, University of Kabianga, Kericho, Kenya; 4 Professional Association of Emergency Surgical Officers (PAESO), Olanchiti Hospital, Adama, Oromia Regional State Ethiopia; 5 Faculty of Medicine, UKMMC, National University of Malaysia, Bangi, Malaysia; 6 Mongolian National University of Medical Sciences, Ulaanbaatar, Mongolia; 7 Department of Health Services, Kenya Clinical Officers Association, County Government Kakamega, Kakamega, Kenya; 8 National Conference of Bar Examiners, Madison, WI United States of America; 9 Royal Ann College of Health, Kumasi, Ghana; 10 Chair, Clinical Officers Council, Nairobi, Kenya

**Keywords:** Accelerated medically trained clinicians, Accreditation, Workforce monitoring, Workforce planning

## Abstract

**Background:**

Shortages and maldistribution of healthcare workers persist despite efforts to increase the number of practitioners. Evidence to support policy planning and decisions is essential. The World Health Organization has proposed National Health Workforce Accounts (NHWA) to facilitate human resource information systems for effective health workforce planning and monitoring. In this study, we report on the accreditation practices for accelerated medically trained clinicians in five countries: Ethiopia, Ghana, Kenya, Malaysia, and Mongolia.

**Method:**

Using open-ended survey responses and document review, information about accreditation practices was classified using NHWA indicators. We examined practices using this framework and further examined the extent to which the indicators were appropriate for this cadre of healthcare providers. We developed a data extraction tool and noted any indicators that were difficult to interpret in the local context.

**Results:**

Accreditation practices in the five countries are generally aligned with the WHO indicators with some exceptions. All countries had standards for pre-service and in-service training. It was difficult to determine the extent to which social accountability and social determinants of health were explicitly part of accreditation practices as this cadre of practitioners evolved out of community health needs. Other areas of discrepancy were interprofessional education and continuing professional development.

**Discussion:**

While it is possible to use NHWA module 3 indicators there are disadvantages as well, at least for accelerated medically trained clinicians. There are aspects of accreditation practices that are not readily coded in the standard definitions used for the indicators. While the indicators provide detailed definitions, some invite social desirability bias and others are not as easily understood by practitioners whose roles continue to evolve and adapt to their health systems.

**Conclusion:**

Regular review and revision of indicators are essential to facilitate uptake of the NHWA for planning and monitoring healthcare providers.

**Supplementary Information:**

The online version contains supplementary material available at 10.1186/s12960-021-00646-4.

## Background

Global shortages of healthcare providers persist [1], despite efforts to increase the number of healthcare workers. The World Health Organization (WHO) has stated that to reach the United Nations’ Millennium Development Goals a mere increase in the number of health care providers is insufficient. Instead, healthcare practitioners must be distributed in a way that makes care accessible, acceptable to the community served, and supported by the health system [2]. To achieve these objectives, it is necessary to ensure that the healthcare providers are ‘fit for purpose’ [3]. This term, ‘fit for purpose’, refers to appropriate education and competencies to meet the needs of local health systems [4, 5]. Many cadres of healthcare workers provide quality care and address issues of maldistribution, improving access due to their knowledge, skills, and competencies. Healthcare systems are challenged in the provision of equitable care and access to underserved populations. In general, policymakers, economists, and researchers have focused efforts on monitoring the number of physicians and nurses in healthcare systems. While doctors and nurses provide important care, shortages and maldistribution continue to be global challenges.

To facilitate the planning of human resource needs, WHO has developed a tool for monitoring inputs and outputs of health systems, the National Health Workforce Accounts (NHWA) [6]. Through the use of indicators, NHWAs are intended to allow the collection of data that improves the monitoring of healthcare workers. The indicators are grouped into ten categories or “modules”. Some indicators directly address the accreditation of educational programmes and the regulation of healthcare workers. These indicators can be used as a framework for consideration of the appropriateness of the education and training of healthcare providers. To be able to manage population needs and healthcare workforce quality and distribution, WHO states that there should be “established accreditation mechanisms for health training institutions” [7]. Accreditation is “a process by which a governmental, parastatal or private body (accreditation agency) evaluates the quality of a higher education institution as a whole, or a specific higher education programme/course, in order to formally recognize it as having met certain predetermined criteria or standards and award a quality label” [8]. Accreditation processes are thought to define the knowledge and skills associated with the profession through evaluation against standards and descriptions of accreditation of nursing and medical education programmes appear frequently in the literature [9–13].

There is a great variation in the education and training of physicians and nurses from country to country, though their roles in the healthcare system are commonly recognized and accepted. This may be because of their established autonomy and visibility to the public [14]. However, there are other cadres, referred to as paramedical practitioners by the International Labour Organization (ILO) or allied health professionals in the United States. According to the International Standard Classification of Occupations, members of this health cadre “provide advisory, diagnostic, curative, and preventive medical services more limited in scope and complexity than medical doctors. They work autonomously or with limited supervision of medical doctors, and apply advanced clinical procedures for treating and preventing diseases, injuries and other physical or mental impairments common to specific communities” [15]. More recently, the ILO has commented that there should be another term in the standards that better distinguish emergency paramedics from others in this occupational classification, titled “accelerated medically trained clinicians” (AMTCs) [16]. The tasks performed by this cadre of health professionals (AMTCs) include diagnostic and advisory duties; advising on public health issues including nutritional methods; improving hygienic and sanitary conditions; dispensing medications; and using preventative and curative medicine. In some locations, the cadre provides surgical and dental care, as well as eye examinations. The scope of care is dependent on training and supervision requirements. AMTCs have provided care with several different titles globally. For example, the first training programmes of physician assistants in Ghana started in the 1960s when they were then called health centre superintendents. They later emerged as medical assistants and are now known as physician assistants [17]. Table 1 provides an overview of cadre titles for five countries. This cadre of health professionals has been responsive to local needs; education and titles vary depending on location. For these reasons, the use of these professionals may not be maximized [5, 16].

**Table 1:** Cadre titles and years of establishment

Country	Cadre title	Year established
Ethiopia	Public health officer	1954
	Integrated emergency surgical officer	2009
Ghana	Physician assistant	1923
Kenya	Clinical officer	1928
Malaysia	Medical assistant/assistant medical officer	1963
Mongolia	Baga Emch/feldsher	1931
Uganda	Clinical officer	1918

Given that much of the literature reports on nurses and physicians, the research reported in this article will focus on the cadre that was identified previously, *accelerated medically trained clinicians* [4]. AMTCs are establishing their profession by improving their visibility as others have in the past; societal recognition of their role and educational requirements including clinical training [14, 18]. Because AMTCs provide care that is fit-for-purpose and are trained in an accelerated manner that is cost-effective [3, 5], understanding quality assurance practices for training programmes and variations is important. The purpose of the current investigation was to provide a view of the state of accreditation based on practices in five countries: Ethiopia, Ghana, Kenya, Malaysia, and Mongolia. Specifically, we examine the current practices in quality assurance for pre-service and in-service training of these accelerated medically trained clinicians using the WHO indicators as a framework. We provide an overview of pre-service and in-service accreditation.

## Methods

We used survey data collected by the World AMTC Network (WAN) using an online form consisting of 52 questions on five topics, including accreditation of pre-service and in-service training (13 questions, 12 open-ended, one categorical; see Additional file 1: Appendix A). WAN members were invited to provide this information and participate in the publication of survey results. Participation was voluntary; a separate recruitment process took place for collaboration on publications. This was a single, cross-sectional survey of the members. We focused our efforts on the analysis of information provided on the accreditation of pre-service and in-service training of AMTCs in Ethiopia, Ghana, Kenya, Malaysia, and Mongolia. These countries were selected as there were representatives who agreed to collaborate on this publication. While survey questions were related to the WHO indicators, we supplemented our review of open-ended responses on accreditation by developing an analysis tool (Additional file 1: Appendix B) that listed the indicators and provided a link between open-ended responses and the indicators. Each of the co-authors completed the analysis tool for their own country using open-ended responses. Indicator information was coded as ‘yes’, ‘no’, or ‘partially’ per the NHWA Guidelines by classifying the match between the open-ended response and the respective indicator. If there was not clear alignment between the indictor and the response, the co-author, as a country representative, completed document review to address that indicator. We report practices by country to identify common and unique themes and to evaluate the use of the WHO NHWA indicators as a framework.

## Results

Using the open-ended survey results and the analysis tool, we summarize our findings in Table 2. Responses were coded to address the indicators and variation in practice. The information in parentheses provides guidance used to interpret open-ended responses. Table 2 shows that all five countries indicate that there are national standards for accreditation of AMTCs (indicator 3–1), that accreditation mechanisms were in place (indicator 3–2), that there are socially accountable standards (indicator 3–3), and those socially accountable standards are effectively implemented (indicator 3–4). In these five countries, there was general agreement on standards (indicator 3–7) and continuing professional development (indicator 3–8).

**Table 2:** National health workforce account indicators by country

Indicator number and name	Ethiopia	Ghana	Kenya	Malaysia	Mongolia
3-01: Standards for the duration and content of education and training (Curriculum standards)	Yes	Yes	Yes	Yes	Yes
3-02: Accreditation mechanisms for education and training institutions and their programmes (Responsibility for accreditation)	Yes	Yes	Yes	Yes	Yes
3-03: Standards for social accountability (Process of the establishment of accreditation)	Yes	Yes	Yes	Yes	Yes
3-04: Standards for social accountability effectively implemented (Accreditation process—stakeholders)	Yes	Yes	Yes	Yes	Yes
3-05: Standards for social determinants of health (Community health component of the curriculum; scope of practice)	Yes	Yes	Yes	Partly	Partly
3-06: Standards for interprofessional education (Ethical issues and professionalism)	Yes	Yes	Yes	Partly	Partly
3-07: Agreement on accreditation standards (Approval, regulation, enforcement)	Yes	Yes	Yes	Yes	Yes
3-08: Continuing professional development (Mandate for professional growth, professional association)	Yes	Yes	Yes	Yes	Yes
3-09: Continuing professional development and specialization training (Medical specialization, the scope of practice, levels of training)	Yes	Yes	Yes	Yes	No

There was more variation in responses for the remaining indicators. Specifically, respondents reported that standards concerned with social determinants of health (indicator 3–5) were partly implemented in Malaysia and Mongolia. This was due to respondents’ interpretation of the role of community health in the standards for training in their respective countries. This was not the case in Ethiopia, Kenya, Ghana, and Malaysia, where community medicine and public health was more clearly the focus of training. For example, in Ethiopia, this cadre is trained to meet the needs of rural and remote communities. The same is true in Kenya, Ghana, Ghana, and Malaysia; the cadre is seen as fit-for-purpose because of accelerated training and the focus on primary care.

Responses varied regarding standards for interprofessional education (indicator 3–6). In Ghana, interprofessional education is embedded in the undergraduate training curriculum of Physician Assistants (PA), whereby PA students study other professional courses and collaborate in teams with other professional students such as nursing, pharmacy, medical laboratory, and medical students during clinical attachments, clerkship, and preceptorship as part of preservice training. The title ‘physician assistant’ refers to three different groups of health professionals [17] trained in the medical model to provide medical and dental care: PA-medical including medical assistants; PA-dental, known as community oral health officers; and PA-anaesthesia, otherwise called nurse anaesthetists.

In Malaysia, interprofessional education (IPE) is not a national education policy, but the concept of IPE is implemented in several higher educational institutions. For example, the National University of Malaysia (UKM), a public university, has implemented IPE in the medical faculty where medical and nursing students study in the same class for certain courses. The primary goal of IPE is to prepare students to work in interprofessional teams and apply this knowledge, skills, and attitudes into their future practice, ultimately providing interprofessional patient care as part of a collaborative team and focusing on improving patient outcomes [19].

In Mongolia, the training of feldshers ended in 2012 in Ulaanbaatar, in 2016 in Dakhan province, in 2017 in Dornogovi province, and in 2018 in Gobi-Altai province, by order of the Ministry of Health. Currently, there are 2410 in active practice. According to the UNDP report, the number of the smallest administrative unit, *bags* increased: in 2007 there were 1539 *bags*, which increased to 1668 in 2019, of which only 965 *bags* (58%) were where feldshers were frontier health specialists providing health and medical services to rural area populations. In Kenya, the evolution of the clinical officer cadre has meant that interprofessional education was essential as there is considerable work with medical officers (physicians) and nurses, particularly as part of in-service training. Currently, there is an independent regulator, the Clinical Officer Council, which may affect the extent to which interprofessional education curricula are used.

For indicator 3–9, there was some variation in the responses. In Ethiopia, standards have been reviewed and revised for additional training and the scope of practice. Recommendations have been submitted to the regulatory authorities. Training for emergency surgical services is also provided in Ethiopia for Integrated Emergency Surgical Officers. In Mongolia, the training of feldshers has ended, but graduates of the programmes continue their professional development in different fields mostly in public health: biostatistics and epidemiology.

In Kenya, clinical officers are currently developing an automated digital platform for the acquisition of new knowledge and updated professional information geared towards improving professional practices across its sub-specialities. In this regard, it is the responsibility of each individual within the profession to acquire the minimum continuing professional development (CPD) points required before the renewal of each individual’s professional practising license. The accounting for CPD points has been done manually since 2016 using signed booklets provided by the Clinical Officers Council. The growing population of professionals and their specialities has necessitated the development of an automated digital platform for this purpose. To do this effectively, the Clinical Officers Council and other stakeholders are currently working with the World Continuing Education Alliance (WCEA) that is already providing the same services to the Kenyan nurses, midwives, and doctors. There are other existing providers of CPD points for clinical officers, other than WCEA. In Ghana, in terms of continuing professional development programmes, PAs can pursue PA speciality and subspecialty programmes in psychiatry and clinical dermatology.

In Malaysia, assistant medical officers/medical assistants are encouraged to pursue continuing education programmes to enhance their knowledge and skills in the respective field of medicine they work. Accordingly, they must earn a minimum of 40 CPD points as a prerequisite for renewing their Annually Practising Certificate (APC). Renewal of APC can be done online using the Business Licensing Electronic Support System. For continuing professional education, accreditation practices varied as well. In some of the countries, respondents thought that this additional training was part of speciality training. Also, the cadre has the opportunity to pursue in-service training for post-basic or advanced diploma programmes (6–12 months) in various areas of specialities such as emergency medicine, orthopaedics, respiratory medicine, critical care, cardiovascular care, renal care, ophthalmology, otorhinolaryngology and many more. The courses are conducted by the Ministry of Health training institutions or private institutions [20].

The open-ended data provided were quite rich and provided insights regarding accreditation processes that go beyond the indicators that are part of the NHWAs. Table 3 provides additional information regarding accreditation of pre-service and in-service training, the establishment of accreditation, and who is responsible for accrediting programmes. The organizations responsible for the accreditation of training for these health professionals are national in origin. For the most part, ministries of education and health are responsible for the accreditation of pre-service training. Based on the responses, these government agencies are responsible for regular curriculum review, coordination of site visits, and establishment of training standards for pre-service training. For in-service training, this responsibility shifted to the licensing authority in the country. Kenya was the exception; this country has a regulatory body (the Clinical Officers Council; COC) that is specific to the Clinical Officers as a cadre.

**Table 3:** Accreditation practices by country

Accreditation characteristic	Ethiopia^1^	Ghana	Kenya	Malaysia	Mongolia
Pre-service accreditation	National accreditation board and professional regulatory body	National accreditation board and the professional regulatory bodies	Clinical Officers Council	Malaysian Qualification Agency (MQA) and Medical Assistants Board (MAB)	National Council for Educational Accreditation
In-service accreditation	Regional health bureau, the regulatory body which gives licenses	Regulatory bodies	Clinical Officers Council	Ministry of Health	National Center for Health Development of the Ministry of Health
Establishment of accreditation	Government	Government	Government	Government	Government
Who is responsible for accreditation?	Ethiopian Food and Drug Authority (EFDA)	National Accreditation Board and the Medical and Dental Council	Clinical Officers Council, Ministry of Health	Medical Assistants Board and Malaysian Qualifications Agency	National Council for Educational Accreditation

^1^Review and update of standards were recently completed

## Discussion

The five countries are interesting contrasts in the role that accreditation has played for AMTCs. Standards are under review in Ethiopia, for all aspects of training. In Mongolia, feldsher training had been around for some time; beginning in the 1934–1935 academic year, medical college and began to prepare feldshers in a 3-year training programme, operated until 1993. Feldshers continue to provide care, particularly in rural and remote areas. In Ghana and Kenya, continuing professional development has meant additional speciality training and expansion in scope. In all five countries, continuing professional development is accredited and monitored. The role of AMTCs in these five countries has existed for some time, which may be why accreditation of pre-service and in-service training are based on best practices, including standards for curriculum and facilities. Pre-service and in-service training are regularly reviewed, ensuring that training meets health needs, and remains fit-for-purpose.

The findings for these five countries show that the NHWA indicators focused on accreditation are met, at least partly. Using the framework to synthesize the information from the survey was not without challenges. Even with extended definitions provided by WHO for the indicators and sub-indicators, there is sufficient room for interpretation that made data coding difficult. In these countries, the AMTC cadre was developed to meet healthcare needs. This meant that social accountability was inherent in the recognition of the cadre and the scope of practice. Most provide primary health care and are the first point of contact for patients, meaning that social determinants of health were a core element in the development of educational programmes. Another area of different interpretation was the conflating of continuing professional development and further specialized training.

The NHWA indicators provided a framework for structuring the survey questions and for synthesizing the responses. However, the definitions for the accreditation module leave room for variation in interpretation. For example, it does not seem that any country would note that there is no requirement to report on standards related to social accountability or that training programmes do not reflect social determinants of health in their curricula. These indicators may identify the extent to which these concepts are universally accepted, but for this cadre, in particular, the context in which these healthcare providers have developed professionally is inherently socially accountable and is concerned with social determinants of health. The respondents from Malaysia and Mongolia reported that social determinants of health indicators were partially met. In these countries, respondents were concerned that while this cadre provides vital primary care services, community health was not explicitly part of the consideration of accreditation standards.

For interprofessional education, the respondents from Malaysia and Mongolia indicated that the indicator was partially met. In Malaysia, this is because interprofessional education is not a national policy, but is implemented in several, but not all, institutions. In Mongolia, this was due to the ending of the training programmes for Baga Emch/feldshers, though the training was conducted in nursing and medical schools. In the five countries that are the focus of the study, interprofessional education is likely as students are often together in early health professions education and proceed to more advanced training based on their academic performance.

NHWA indicators provided a framework to consider the accreditation practices in these five countries. The intention of the collection and monitoring of this data is to provide an infrastructure to assist in meeting universal health coverage. The use of these indicators is meant to be used for national monitoring and planning of the health workforce [6]. For the current investigation, we examined the indicators in module 3 for advanced medically trained clinicians. As mentioned earlier, this cadre may consist of different types of healthcare professionals, with varying practice characteristics. While there is advocacy for reconsidering this categorization, the current plan may not permit accurate data for monitoring and planning. One issue is the dominance of certain professions in the framework as these professions are seen as a “set of easily identifiable occupations with a high degree of comparability between countries, ensuring a harmonized monitoring of the health workforce” [6]. While standards improve comparability, if the goal is to provide countries with information to determine the extent to which there is a sufficient supply of healthcare workers with the right mix of skills, then it is important to consider the context. The use of the indicators, even with the detailed explanations, was still difficult to adapt to report the accreditation practices in the five countries.

In the countries in this study, the development of the profession came out of a need for healthcare services not addressed by other health professionals. As various cadres develop accelerated training to acquire fit-for-purpose skills, it will be important to consider several factors that affect the determination of the adequacy of the health workforce. The accelerated medically trained clinicians in these countries continue to serve in locations where there are shortages of physicians and nurses. In some countries, the development of specialization for AMTCs has been cost-effective and has allowed the expansion of services [21]. It is important to determine how this information can be collected, particularly in countries where this cadre exists, but has not yet gained recognition. This lack of formal recognition could result in what has been referred to as “wastage”, where workers with the appropriate skill mix are not used, or underutilized [22]. Contribution to the data by different organizations and agencies and increased understanding of the scope of practice for healthcare workers is essential should these indicators be used as planned. The definitions and frameworks employed need to support efforts to clearly define training as well as the scope of practice. Implementation of the NHWA indicators should support clarity in the various cadres providing healthcare, though research has shown that policy documents may not reflect the contributions of this cadre [23, 24].

While the findings in the study provide insight into the accreditation practices for the education of AMTCs in these five countries, it is not without limitations. First, in reviewing the survey results, we focused on responding countries and then focused on those countries where there were co-authors to assist in aligning responses with the indicators. This could represent a biased sample since the focus is on those countries willing to discuss accreditation practices. Second, there may be other countries where AMTC training is accredited but no one from that country responded to the survey. Third, the survey was distributed to members of the World AMTC Network. While membership is free, it is not possible to determine whether others would have provided additional information about accreditation practices.

## Conclusion

Despite these limitations, the results show that while it is possible to use NHWA Module 3 indicators to categorize accreditation practices, there are disadvantages. The indicators, while standardized, overlook important contextual features that provide valuable information on the contributions of AMTCs to their respective country’s workforces. Ensuring the inclusion of all workers will ensure that a more realistic view of healthcare provision is monitored. In particular, it is important to accurately capture information for those providers whose roles continue to evolve to be fit-for-purpose. Regular review of the indicators and their definitions and input from a wide variety of stakeholders may increase the use of the NHWA for planning and monitoring.

## Supplementary Information

**Additional file 1.** Appendices A and B.

## Data Availability

The datasets used and/or analysed during the current study are available from the corresponding author on reasonable request.

## Abbreviations

AMTC: Accelerated medically trained clinicians
APC: Annually Practising Certificate
ILO: International Labour Organization
IPE: Interprofessional education
NHWA: National Health Workforce Account
PA: Physician assistant
UKM: National University of Malaysia
WAN: World AMTC Network
WCEA: World Continuing Education Alliance
WHO: World Health Organization
