# Supplementary material for: Global accreditation practices for accelerated medically trained clinicians: a view of five countries
Source: Hum Resour Health. 2021 Sep 14;19:110. doi: 10.1186/s12960-021-00646-4 (PMC8438892; doi:10.1186/s12960-021-00646-4)
Supplement: Supplementary file 1 — Additional file 1. Appendices A and B. [file 12960_2021_646_MOESM1_ESM.docx]

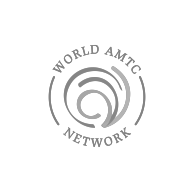


**WELCOME**

**Dear World AMTC Network Member,**

**We are excited to reach out to you to share your expertise for a series of articles highlighting accelerated medically trained clinicians around the world.**

**All survey completers will be acknowledged, in the article that their contribution is used. We are eager to have you engage as *co-authors*. This entails helping organize, develop, write, serve as a lead of sections possibly, etc.**

**The series will have the overarching theme of how AMTC cadres globally are meeting population needs for UHC - through the specific lenses of accreditation and regulation.**

**The five chapter themes are:**

**1. history /evolution of the profession in your country**

**2. pre-service and in-service training (students and practicing clinician -CPD)**

**3. scope of practice/ expanded scope of practice (specialty care/task sharing - training in addition to the original training)**

**4. regulatory council process (body that regulates your professional clinical practice, examples: Allied Health Professional Council, Clinical Officer Council, Medical and Dental Council, etc) 5. summary article - comparing and contrasting the prior 4 chapters**

**You will be asked to choose one of the four main theme areas to answer questions on.** T**hemes will help determine which areas you would like to have represented for your cadre/country, have data for, and how you would like to engage in these research publications.**

**As we assess the survey results, we will call for meetings, and work with you in teams to write these articles.**

**Thank you for participating in our survey. Your feedback is important.**


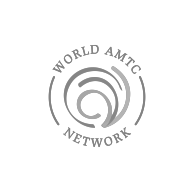
n and Regulation Member Survey

1. Please choose which country you are working in

|  |
| --- |

2. What is your primary AMTC role at this time

|  |
| --- |

3. Please choose ONE chapter theme - you will be directed to answer specific questions within that theme. Please pick a theme you are most familiar with.


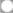
History / Evolution of the Profession


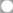
Pre-Service and In-Service Training (student and practicing clinician)


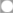
Expanded Scope of Practice (specialty care or task sharing)


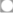
Regulatory Council Process (bodies that oversee the practice)


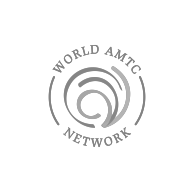
Accreditation and Regulation Member Survey

**Pre-service and In-service Training**

**Pre-service: student training**

**In-Service: CPD (continuing professional development), CME (continuing medical education, etc)**

11. How has Accreditation *impacted* the training /curriculum - pre-service/in-service of the profession?

|  |
| --- |

12. How has Regulation *impacted* the training /curriculum - pre-service/in-service of the profession?

|  |
| --- |

13. How has the training/curriculum - pre-service/in-service *impacted* Accreditation?

|  |
| --- |

14. How has the training/curriculum - pre-service/in-service *impacted* Regulation?

|  |
| --- |

15. How is your pre-service training accredited?

|  |
| --- |

16. How is your in-service training accredited? (continuing professional development, continuing medical education, etc)

|  |
| --- |

17. How was this accreditation established?

|  |
| --- |

18. Who are the accreditors?

|  |
| --- |

19. Where are the accreditors housed? (Ministry of Education, Ministry of Health, separate organization/council, etc)

|  |
| --- |

20. What is accredited?


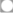
individual training programs


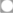
overall curriculum


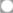
institution where the training program is housed


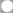
Other (please specify)

|  |
| --- |


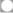
None of the above

21. How is accreditation: determined

Are there set standards?

|  |
| --- |

if yes- what are they

22. What drives change in Accreditation?

|  |
| --- |

23. What is the process to implement change in Accreditation?

|  |
| --- |

**Appendix B: Analysis Tool Accreditation, Education and Training – Accelerated Medically Trained Clinician Survey**

Country:

Name of the AMTC

Continent:

| **Module** | **Name** | **Indicator Name** | **YES** | **NO** | **PARTLY** |
| --- | --- | --- | --- | --- | --- |
| 3-01 | Standards for the duration and content of education and training  **Curriculum** | Existence of national and/or sub-national standard on the duration and content of health workforce education and training  **Curriculum** |  |  |  |
| 3-02 | Accreditation mechanisms for education and training institutions and their programmes  **Responsibility for accreditation** | Existence of national and/or sub-national mechanisms for accreditation of health workforce education and training institutions and their programmes  **laws/policy/written mechanism** |  |  |  |
| 3-03 | Standards for social accountability  **Process of establishment** | Existence of national and/or sub-national standards for social accountability in accreditation mechanisms  **Composition of the regulatory Bodies** |  |  |  |
| 3-04 | Standards for social accountability effectively implemented  **Accreditation process –among the stakeholders** | National and/or sub-national standards for social accountability in accreditation mechanisms are effectively implemented  **Stakeholder involvement** |  |  |  |
| 3-05 | Standards for social determinants of health  **The community health component of the curriculum scope of practice** | Existence of national and/or sub-national standards for the social determinants of health in accreditation mechanisms  **community health component in the curriculum** |  |  |  |
| 3-06 | Standards for interprofessional education  **Ethical issues and professionalism** | Existence of national and/or sub-national standards for interprofessional education in accreditation mechanisms  **Interprofessional issues** |  |  |  |
| 3-07 | Agreement on accreditation standards  **Approval regulation and enforcement** | Existence of cooperation between health workforce education and training institutions and regulatory bodies to agree on accreditation standards  **Approval /Certification/written agreement** |  |  |  |
| 3-08 | Continuing professional development  **Professional association and mandate for professional growth** | Existence of national systems for continuing professional development  **Compulsory CPD and enforcement mechanism and licensing** |  |  |  |
| 3-09 | Continuing Professional Development **specialization training**  **Medical specialization, the scope of practice, levels of training** | Existence of in-service training as an element of national education plans for the health workforce  **Specialization training and duration of training including the scope of training**  **Advanced Diploma**  **Postgraduate Diploma**  **Master in Clinical Medicine & others** |  |  |  |

Source: Adapted from WHO NHWA Handbook

Developed by Erick Kizito Wanyama
